# LEVERAGING SOURCES TO ENHANCE INTERPROFESSIONAL GERIATRIC EDUCATION

**DOI:** 10.1093/geroni/igae098.1086

**Published:** 2024-12-31

**Authors:** Gina Tucker-Roghi, Alissa Cooney, Rajean Moone, Christina Cauble

**Affiliations:** Dominican University of California, San Rafael, California, United States; UT Health San Antonio, San Antonio, Texas, United States; University of Minnesota, St. Paul, Minnesota, United States; University of Minnesota Medical School, Minneapolis, Minnesota, United States

## Abstract

Geriatrics is a highly interdisciplinary field, and methods to enhance geriatric education need to be equally interdisciplinary. A critical step is identifying, engaging, and mobilizing a wide array of stakeholders and educational resources to enhance geriatrics integration across various medical disciplines. This session will delve into methodologies used for discovering stakeholders in geriatrics within a local institution and the community at large, emphasizing the creation of collaborative networks that promote geriatrics principles in educational and clinical settings. Participants will explore the experiences of geriatricians and interprofessional geriatrics specialists at academic institutions leveraging their knowledge and resources at their institutions and local communities. This session aims to provide insights into effective strategies for recognizing and collaborating with contributors to geriatric care, even those who may not see themselves as specialists. We will explore how geriatric educational leaders can reduce stigma and utilize stealth and speed to maximize local resources and age-friendly initiatives.Through engaging with and mobilizing a diverse network of stakeholders, educators can foster a more robust integration of geriatric principles, ensuring that healthcare professionals across specialties are well-prepared to meet the unique needs of the aging population. The session will offer actionable steps for attendees to engage with institutional and community stakeholders, turning them into vital sources of geriatric expertise and fostering a more inclusive and comprehensive approach to geriatric education and practice.

